# Handling the data management needs of high-throughput sequencing data: SpeedGene, a compression algorithm for the efficient storage of genetic data

**DOI:** 10.1186/1471-2105-13-100

**Published:** 2012-05-16

**Authors:** Dandi Qiao, Wai-Ki Yip, Christoph Lange

**Affiliations:** 1 Department of Biostatistics, Harvard School of Public Health, 677 Huntington Avenue, Boston, Massachusetts, USA; 2 Institute for Genomic Mathematics, University of Bonn, Bonn, Germany; 3 German Center for Neurodegenerative Diseases (DZNE), Bonn, Germany

## Abstract

**Background:**

As Next-Generation Sequencing data becomes available, existing hardware environments do not provide sufficient storage space and computational power to store and process the data due to their enormous size. This is and will be a frequent problem that is encountered everyday by researchers who are working on genetic data. There are some options available for compressing and storing such data, such as general-purpose compression software, PBAT/PLINK binary format, etc. However, these currently available methods either do not offer sufficient compression rates, or require a great amount of CPU time for decompression and loading every time the data is accessed.

**Results:**

Here, we propose a novel and simple algorithm for storing such sequencing data. We show that, the compression factor of the algorithm ranges from 16 to several hundreds, which potentially allows SNP data of hundreds of Gigabytes to be stored in hundreds of Megabytes. We provide a C++ implementation of the algorithm, which supports direct loading and parallel loading of the compressed format without requiring extra time for decompression. By applying the algorithm to simulated and real datasets, we show that the algorithm gives greater compression rate than the commonly used compression methods, and the data-loading process takes less time. Also, The C++ library provides direct-data-retrieving functions, which allows the compressed information to be easily accessed by other C++ programs.

**Conclusions:**

The SpeedGene algorithm enables the storage and the analysis of next generation sequencing data in current hardware environment, making system upgrades unnecessary.

## Background

As the influx of high-throughput sequencing data [1-3] is imminent, the data management requirements for the analysis packages have changed fundamentally. While, during the days of candidate gene analysis and linkage analysis,”only” up to several thousands of genetic loci had to be stored and loaded into the analysis packages, current Genome-wide Association studies (GWAS) provide genetic information on several millions of genetic loci. Thus, the typical size of a dataset containing mostly common variants is about 1 to 30 Gigabytes. For high-throughput sequencing studies, the number of genetic loci genotyped increases by several magnitudes, and the file size of such sequencing data can be up to several Terabytes. For such large files, the loading process can take up to few hours without counting the time for analysis. This results in great waste of disk space and computation time, which is a problem that is encountered routinely.

One possible solution is to use the general-purpose compression software, such as Gzip and BGZip. However, such compression software is not designed specifically for genetic data and its analysis, so the compression rate is relatively low and decompression is always needed before accessing the data. Better solutions have been proposed. PLINK and PBAT, which are free whole-genome association analysis toolsets, have introduced Binary PED formats [4,5]. This format ensures that only 2 Bits are required for storing the information of one genotype. It is the most popular compression format used in GWAS. However, the compression rate is not sufficient for massive datasets generated nowadays as their compressed datasets could still occupy several Gigabytes of the disk space. In recent years, sophisticated compression techniques designed specifically for sequencing data have been proposed. For example, DNAzip [6] introduced the idea of storing only the difference between one individual genome data and a reference genome. However, such algorithms suffer the large overhead for storing the reference genome. Also, they require substantial CPU-time for decompression.

We propose here a simple and efficient algorithm to store large datasets containing SNP data of multiple samples. We show that our algorithm always works better than the compression algorithm implemented in PLINK or PBAT and provides excellent compression rate for sequencing data. Also, the compressed data structure provides the potential for efficient implementation of permutation methods and does not require any overhead CPU-time for decompression. We have implemented the algorithm in the GPL licensed C++ library: SpeedGene. We show that it takes much less time for loading the compressed files than PLINK using our library. In addition, Our C++ implementation supports parallel loading of the genetic information, which further decreases the loading time as the number of parallel jobs increases. The version 1.0 of the SpeedGene library is available at http://people.hsph.harvard.edu/∼ dqiao/SpeedGene.html together with detailed instructions and examples.

## Methods

### The LINKAGE/PLINK data format

The LINKAGE or PLINK data format is a commonly used data format for storing SNP data in Genome-Wide Association studies. Data files in this format are called pedigree files and have”.ped” as the suffix. This format can be converted from or to the VCF format used in 1000 Genome Project using VCFtools [7]. The SpeedGene library currently only recognizes pedigree files in the LINKAGE/PLINK format, but the algorithm can be implemented for compressing SNP data in the VCF format. The VCF format requires the same amount of disk space for each genotype (4 Bytes) as the LINKAGE/PLINK format, so the compression rate of this algorithm applying on VCF files should be similar to the compression rate for pedigree files. Note that VCF files may contain other informations such as Indels and whether the genotype is phased or unphased, which could not be incorporated into the LINKAGE format. However, since SNP data are very commonly used genetic data in association studies and takes the most disk space, efficient storage of the SNP data could still save a lot of resources. In the demonstration of the algorithm and the examples below, we use the LINKAGE/PLINK format as the input format.

**Figure 1:**
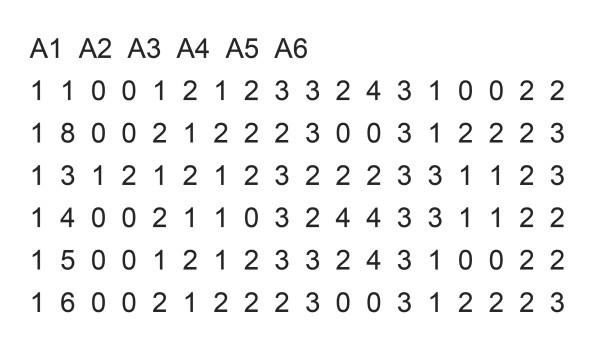
**An example of the LINKAGE format A toy example of a pedigree file in the LINKAGE format.** The first line contains the marker names. Starting from the second line, each line contains the pedigree and genetic information for each individual. The first six columns indicate the subject’s pedigree ID, subject ID, father ID, mother ID, sex and affection status. The other columns contain the genetic data.

Any pedigree file in the LINKAGE format has the same structure, a toy example is shown in Figure 1. The first line contains the marker names, separated by a space character. Starting from the second line, each line includes pedigree and genetic information for each individual. The first six columns of these lines specify each individual’s pedigree information in the order of pedigree ID, subject ID, father ID, mother ID, sex, and affection status. Subject ID must be unique within one’s family. Father and mother ID could be 0 if this information is unknown, e.g. population-based study of unrelated subjects. Sex is 1 for male and 2 for female. Affection status is 1 if the subject is unaffected, 2 if affected, and 0 if the status is unknown. The other columns contain the genetic data for each individual, separated by a space between each marker. Two columns are required to represent the information for two alleles, separated by a space. The allele in- formation is coded using 0 to 4 where 1 = A, 2 = C, 3 = G, T = 4 and 0 represents missing allele information.

### The SpeedGene algorithm

The SpeedGene algorithm consists of three different sub-algorithms, which are selected by SpeedGene based on the minor allele frequency (MAF) of the genetic locus to be stored. The space needed for the compressed data is computed for the sub-algorithms beforehand. The SpeedGene algorithm then selects the best procedure among the three compression methods. The first sub-algorithm is based on the binary format implemented in PLINK and PBAT. It utilizes the fact that the marker information of each marker can be represented using a 2-digit binary number. The second sub-algorithm uses subject indices to indicate heterogeneous, homogeneous and missing genotypes. The third sub-algorithm uses binary digits to indicate heterogeneous genotype and subject indices to indicate homozygous and missing genotypes. A feature of all three compression methods is that the required memory space for storage can be computed prior to compression. Thereby, the SpeedGene algorithm is able to select the optimal method before compressing the data. The three sub-algorithms are described in detail in the following sections.

#### Sub-algorithm I: compression using binary encoding

For any pedigree file, we assume that there are only bi-allelic markers in the file. For any allele of a marker, an individual may only have 0, 1 or 2 of this allele. Also, the allele information can be missing for any individual at any marker. Thus, the marker information can be transformed into the number of copies of a particular allele. It could be 0,1,2, or missing and could be converted to a 2-digit binary number. In the compression process, we find the minor alleles at each marker and use 00, 01, 10 to represent zero, one or two copies of the minor allele at one marker. 11 indicate that the genetic information is missing at this marker for the individual. Thus, one genotype in the original file can be converted into two binary digits, which is 2 Bits on disk space. Four of such 2-digit binary number is 8 Bits, which equals 1 Byte. Therefore, the genetic information of four markers for one individual can be converted into 1 Byte in a binary file. This binary encoding is similar to the binary format used in PLINK [5] or PBAT [4].

**Figure 2:**
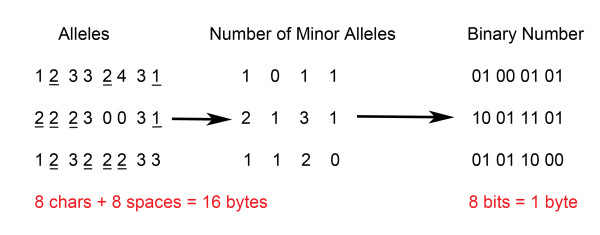
**Application of Sub-algorithm I to the toy example.** Genetic information of the first four markers for the first three individuals in the toy example is extracted here to demonstrate the sub-algorithm I. Each row represents the four genotypes of one individual. The minor alleles for the four markers are assumed to be 2 2 2 1 respectively, and are underlined. Genotype 0 0 represents missing genotypes in the original dataset, which is converted to 3 to indicate missing genotypes.

Based on this conversion method, we can compress the genetic information in the pedigree file into a much smaller binary file. As we have seen in the example (Figure 2), the genetic information for four genotypes occupies 16 Bytes in the original pedigree file, and it is converted to only 1 Byte in the compressed file, which could save up to a factor of sixteen on the disk space. If there are n subjects in the dataset, the storage requirement for compressing n genotypes for one marker using this algorithm is given by

(1) $$\left\lceil 2*n/8\right\rceil \;Bytes$$

For the assessment of the performance of the proposed SpeedGene algorithm, we will use the LINKAGE/PLINK format and the binary-encoding algorithm described above as the standard approach to which the SpeedGene algorithm will be compared.

#### Sub-algorithm II: compression using subject indices

With the binary-encoding algorithm described above, the genetic information of any marker in one dataset is compressed to the same size since the compression algorithm does not depend on the frequency of each genotype. As shown in the results section, the performance of the binary compression is the best we can achieve when the variants are relatively common (MAF > 30%). However, for SNPs with small MAF, only a few subjects have the heterozygous genotype and, even fewer, have the rare homozygous genotype. Thus, it is wasting disk space if the genetic information for all the subjects is recorded, especially for the subjects with the common homozygous genotypes which is by far the most frequent genotype. Therefore, we can utilize this feature of SNPs with small MAF, and record only the indices of the subjects with the missing, heterozygous or rare homozygous genotypes for the SNP. The common homozygous genotype is the default genotype. Since most of the SNPs of the human genome have small MAF [1], the improvements of this approach is substantial compared to the binary-encoding algorithm in the last section.

Specifically, suppose we have n subjects in the data, then we need $\left\lceil \log_{2}\left(n\right)\right\rceil$ binary digits in order to record the index of any subject. First, the number of the rare homozygous, the heterozygous and the missing genotypes are counted. This information is used to calculate the compressed size and determine whether Sub-algorithm II should be used for the SNP. If Sub-algorithm II requires the smallest amount of memory, SpeedGene will use Sub-algorithm II for the compression of the genetic data for the SNP. The indices of the subjects with the homozygous, heterozygous and missing genotypes are transformed into binary digits and are written into the binary file afterwards. Since the number of subjects with each genotype varies, the counts, each requires $\left\lceil \log_{2}\left(n\right)\right\rceil$ Bits on the disk space, are written to the file before the indices of the subjects are outputted to the file. Thus, the storage requirement for compressing n genotypes for one marker using this algorithm is given by

(2) $$\left\lceil \left\lceil \log_{2}\left(n\right)\right\rceil *\left(\#Homo+1+\#Heter+1+\#Missing+1\right)/8\right\rceil \;Bytes$$

where #Missing denotes the number of subjects with the missing genotype, #Homo denotes the number of subjects with the rare homozygous genotype, and #Heter denotes the number of subjects with the heterozygous genotype.

#### Sub-algorithm III: compression using binary encoding and subject indices

As we will see in the next section, Sub-algorithm II works best for SNPs with very small MAF, but performs worse than Sub-algorithm I for more common SNPs (MAF > 0.3). However, by combining Sub-algorithm I and II, we can create a hybrid approach that performs better than Sub-algorithm I and II for SNPs whose MAFs are somewhere between uncommon and very common.

Since the heterozygous genotype is more common for genetic loci that are in the range between uncommon and very common (0.05 ≤ MAF ≤ 0.3), recording the heterozygous genotype by the indices of subjects is not very efficient. Instead we use a binary number of n digits to indicate the subjects with the heterozygous genotype, where n is the number of subjects in the dataset. If subject i has the heterozygous genotype for the SNP, 1 is put at position i instead of 0. Beside this, the indices of subjects with the missing and homozygous minor allele genotypes are recorded in the same way as in Sub-algorithm II. The storage requirement of the marker information for n samples using this algorithm is given by

(3) $$\left\lceil \;\left(\left(\#Homo+1+\#Mis\sin g+1\right)*\left\lceil \log_{2}\left(n\right)\right\rceil +n\right)/8\right\rceil \;Bytes$$

where #Homo denotes the number of subjects with the rare homozygous genotype and #Missing denotes the number of subjects with the missing genotype for the SNP.

For Sub-algorithm II and III, since the indices of the heterozygous and homozygous genotypes are stored for each marker, this compressed data structure makes computation for permutation methods much convenient.

## Results and discussion

### Performance comparison of sub-algorithms

**Figure 3:**
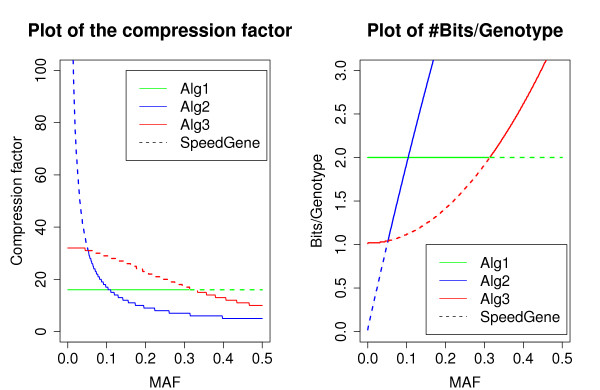
**Plots of the compression rate vs. MAF.** In the left plot, the compression factors of the three sub-algorithms are plotted against different MAF levels. In the right plot, the number of Bits needed for storing one genotype is plotted against different MAF levels. 1000 genotypes are simulated for one SNP at each MAF level. The space needed to store this information for one SNP in the LINKAGE/PLINK format is 4000 Bytes. The compression factor is the number of times by which the compressed file is smaller than the original file size (4000 Bytes).

The SpeedGene algorithm selects for each genetic locus the optimal algorithm in terms of storage space (1–3) among the three sub-algorithms as described in the methods section. To assess the performance of the SpeedGene algorithm, we compare it with the standard LINKAGE/PLINK format and the PLINK/PBAT compression algorithm. The efficiency of the SpeedGene algorithm depends on two factors, the genotype frequency of the genetic locus and the number of subjects included in the dataset. Assuming Hardy-Weinberg equilibrium, the first plot of Figure 3 gives a plot of the compression factor of the three sub-algorithms versus different MAFs for a dataset of 1000 subjects. The second plot shows the number of Bits needed per genotype for storing the genotype information of 1000 subjects at different MAF values. The dashed line provides the performance for the SpeedGene algorithm which is based on the allele frequency and formulas 1, 2 and 3 to select the optimal compression procedure among Sub-algorithm I-III.

As in the plot, approximately, SpeedGene always achieves a compression factor of 16 compared to the standard LINKAGE format for MAF > 0.3 for which Sub-algorithm I is used. SpeedGene accomplishes a compression factor of 16 up to 30 compared to the LINKAGE/PLINK format for 0.05 ≤ MAF ≤ 0.3 for which Sub-algorithm II is selected. For rare and uncommon alleles (MAF < 0.05), a compression factor of at least 30 compared to the LINKAGE format is realized. With smaller MAFs, the compression factor increases rapidly. Equivalently, 2 Bits per genotype would be needed for MAF > 0.3, about 1.0 to 2.0 Bits per genotype for 0.05 ≤ MAF ≤ 0.3, and less than 1 Bit per genotype is needed for MAF < 0.05.

**Figure 4:**
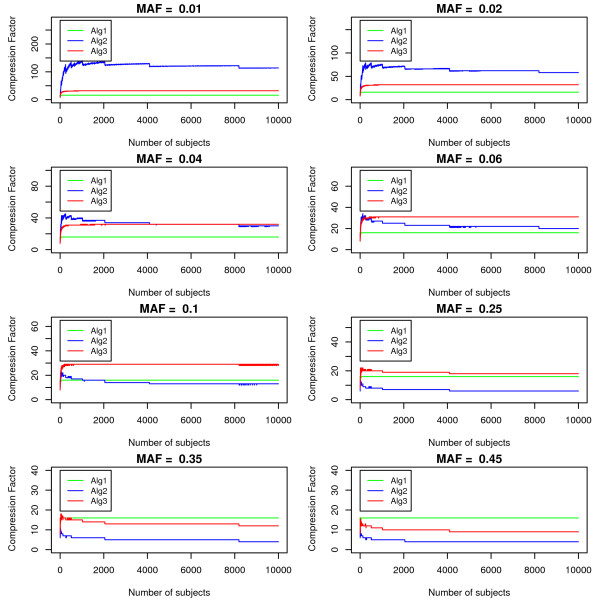
**Plots of the compression rate vs. number of subjects.** The compression factors of the three sub-algorithms are plotted against the number of subjects include in one dataset at eight different MAF levels, which are 0.01, 0.02, 0.04, 0.06, 0.1, 0.25, 0.35 and 0.45. The datasets we considered included at least 100 subjects and contain only one marker with the specified MAF level.

The performance of the algorithms also depends on the number of subjects in the dataset. Figure 4 shows the compression factor of the algorithms for one marker for different number of subjects, at eight MAF levels. Generally, the compression factor decreases slightly as the number of subjects included increases, but is mostly constant over the range of number of subjects we have considered for different values of MAF. In addition to that, the plots give us similar information as the plots above. For example, for SNP with MAF = 0.01, Sub-algorithm II is able to compress the genetic information by a factor of at least 100, which is much better than Sub-algorithm I and III. Thus, MAF is the most influential factor in determining which algorithm is the optimal method among the three sub-algorithms.

### The C++ library implementation

We have implemented the algorithm in a C++ library called SpeedGene. There are two classes in the SpeedGene library. The first one is the Comp class, which is responsible for compressing a pedigree file in the LINKAGE/PLINK format into a text file that contains the subject information and a binary file that contains the genetic information. The binary file is not human-readable and can only be used by the second class in our library. The compression process requires two scans of the pedigree file to avoid storing all the marker information before compression, which would take a great amount of memory space. The second class is the LoadComp class. As its name suggested, it is responsible for loading the compressed files into the memory, and for processing queries from the user. It provides an option to load the entire pedigree file or to load a section of the file. This partial-loading function ensures that only necessary information is loaded for the jobs that are running in parallel, which greatly decreases the loading time. Moreover, the public functions provided by the library allow the user to retrieve any information stored in the original file. This C++ library makes it straightforward for users to incorporate it into their own programs whereas other existing libraries do not offer such capability.

### Performance

#### Compression rate

We evaluated the performance of the SpeedGene algorithm on two rare variants datasets. We simulated two datasets with 1000 subjects from the Wright’s distribution [8], which is $f\left(p\right)=cp^{\beta s-1}{\left(1-p\right)}^{\beta n-1}e^{\sigma }{}^{\left(1-p\right)}$, where the scaled mutation rates βs = 0.001, βn = βs/3, the selection rate σ = 12, and c is a normalizing constant. Table 1 below shows the compressed file size for the simulated data. For sequencing data, the optimal algorithm is Sub-algorithm II for most of the SNPs. Thus, SpeedGene is able to achieve a large compression rate. In the simulated data, the compression factor is approximately 200, which is equivalent to 0.16 Bits per genotype, whereas 2 Bits per genotype is required by PLINK or PBAT. Gzip seems to perform much better on rare variant data than on common variant data, however, such general-purpose software takes extra time to decompress the files before loading them into the memory. We have also extrapolated the approximate file size if DNAzip is used [6]. According to the paper, each SNP for one person requires slightly less than 1 Byte per SNP for storage and it requires a reference hu- man genome (∼ 3 Gigabytes) and a reference SNP map (∼ 1.2 Gigabytes) to retrieve the entire genome data.

**Table 1:** Performance of the SpeedGene algorithm on the simulated datasets

SNPs	Size	PLINK	Gzip	SpeedGene	DNAzip (Extrapolated)	Avg MAF
1 million	3.731 GB	238 MB	22 MB	18 MB	16 MB + ∼ 4.2 GB reference	0.004944
30 million	112 GB	6.985 GB	592 MB	534 MB	310 MB + ∼ 4.2 GB reference	0.004228

Compressed file sizes of the simulated datasets using PLINK, Gzip, SpeedGene and DNAzip. Each dataset contains 1000 subjects.

We also applied these methods to two real datasets. One dataset contains the genotype data from the Framingham Heart Study (FHS), which includes 6956 subjects and 340,444 SNPs. The other dataset is obtained from the COPDgene study on patients with Chronic Obstructive Pulmonary Disease (COPD). It includes 257 subjects with 162757 SNPs over the human genome and 77% of the SNPs in this sequencing data have a MAF ≤ 5%. The original file size and the compressed file sizes using different compression methods are shown in Table 2. For the FHS dataset, since that most of the SNPs are common, the compression rate of SpeedGene is just slightly greater than that of PLINK. Gzip gives a much lower compression ratio of 6 here, as for most common variant datasets. The COPDgene sequence data contains mostly rare variants, but still includes some common variants, so we observe a much higher compression rate with SpeedGene than with PLINK and Gzip.

**Table 2:** Performance of the SpeedGene algorithm on two real datasets

Dataset	Size	PLINK	Gzip	SpeedGene	Avg MAF
FHS	8.822 GB	564.6 MB	1.400 GB	460 MB	0.238637
COPDgene	161 MB	10.1 MB	20.5 MB	3.6 MB	0.057327

File sizes of the FHS dataset and COPDgene dataset, compressed using PLINK, SpeedGene and Gzip.

#### Loading time

The time for loading the compressed datasets using SpeedGene and PLINK on a 2.35 GHz AMD Opteron CPU with 128 GB of RAM is shown in Table 3 below. The time to load the entire file using SpeedGene is less than half of the time needed by PLINK for the simulated datasets. If the analysis is ran in parallel, the loading time using SpeedGene is decreased further as the number of jobs ran in parallel increases. For example, if we are loading 1/10 of the dataset with 30 million SNPs in each parallel job, the loading time needed by SpeedGene is 1.8 minute.

**Table 3:** Time needed to load the compressed dataset

Number of SNPs	Loading time (SpeedGene)	Loading time (PLINK)
1 million	26 sec	56 sec
30 million	11 min	29 min

The CPU time needed for loading the two compressed files using SpeedGene and PLINK on a 2.35 GHz AMD Opteron CPU with 128 GB of RAM.

## Conclusions

To tackle the problem of large file sizes and long loading times of genetic data, we have developed a new compression algorithm - SpeedGene. The algorithm selects the optimal approach among three methods in terms of the required disk space. We have shown that the algorithm always works better than the compression algorithms provided by PBAT and PLINK, and can reach a compression factor of sixteen up to few hundreds. Especially for sequencing data with mostly rare variants, the algorithm is able to compress files of hundreds of Gigabyte to hundreds of Megabytes. Similar compression rate can be reached for the VCF files containing SNP data. In addition, the compressed data structure requires no extra time for decom- pression and could reduce a large amount of computation time for performing permutations on the genotypes.

A C++ implementation of the SpeedGene algorithm is provided and an integration in R is ongoing, but the algorithm could be implemented easily for other data formats and using other programming languages. The SpeedGene library utilizes the structure of the compressed data and enables direct loading of the genotype data into the memory. Moreover, the functions in the LoadComp class of this library allow the user to flexibly retrieve any specified subject or genetic information from the compressed dataset. Furthermore, user-friendly parallel-loading function is supported, which in result shortens the loading time greatly when parallel jobs are dispatched in clusters.

To fully utilize the compression algorithm, it needs to be incorporated into other analysis software for association studies, where the genetic information can be loaded using the library and directly sent for analysis in the software. For example, we are planning to include this binary format as one of the standard input format in NPBAT, which is an interactive software for the analysis of population based genetic association studies. Such incorporation would require additional efforts, but with the gain of much more disk space and shorter loading time, it will be beneficial in the long run.

## Competing interests

The authors declare that they have no competing interests.

## Authors’ contributions

DQ and CL conceived of the project idea, developed the algorithm and wrote the manuscript. DQ implemented the algorithm in the C++ library. WKY was responsible for preparing the testing datasets. All authors read and approved the final manuscript.

## References

[B1] The 1000 Genome Project Consortium A map of human genome variation from population-scale se- quencing Nature 2010 467 1061 1073 10.1038/nature09534 20981092 PMC3042601

[B2] Bansal V Libiger O Torkamani A Schork N Statistical analysis strategies for association studies involv- ing rare variants Nat Rev Genet 2010 11 773 785 20940738 10.1038/nrg2867 PMC3743540

[B3] Metzker ML Sequencing technologies - the next generation Nat Rev Genet 2010 11 31 46 10.1038/nrg2626 19997069

[B4] Lange C Dawn D Edwin KS Scott TW Nan ML PBAT: tools for family-based association studies Am J Hum Genet 2004 74 2 367 369 10.1086/381563 14740322 PMC1181934

[B5] Purcell S Neale B Todd-Brown K Thomas L Ferreira MAR Bender D Maller J Sklar P de Bakker PIW Daly MJ Sham PC PLINK: a tool set for whole-genome association and population-based linkage analysesl Am J Hum Genet 2007 81 3 559 575 10.1086/519795 17701901 PMC1950838

[B6] Christley S Lu Y Li C Xie X Human genomes as email attachements Bioinformatics 2009 25 274 275 10.1093/bioinformatics/btn582 18996942

[B7] Danecek P Auton A Abecasis G Albers CA Banks E DePristo MA Handsaker R Lunter G Marth G Sherry ST McVean G Durbin R 1000 Genomes Project Analysis Group The variant call format and VCFtools Bioinformatics 2011 27 2156 2158 10.1093/bioinformatics/btr330 21653522 PMC3137218

[B8] Wright S Jepson GL, Simpson GG, Mayr E Adaptation and selection Genetics, paleontology and evolution 1949 Princeton University Press, Princeton, New Jersey 365 389

